# *Cheonggukjang* Attenuates Lipid Dysmetabolism by Modulating Gut Microbiota-Derived SCFA Production and G-Protein-Coupled Receptor Levels in White Adipose Tissue

**DOI:** 10.3390/foods15152653

**Published:** 2026-07-28

**Authors:** Su-Bin Lee, Do-Youn Jeong, Youngmi Lee, Ok-Kyung Kim, Anna Han

**Affiliations:** 1 Department of Food Science and Human Nutrition, Jeonbuk National University, Jeonju 54896, Republic of Korea; 2 Microbial Institute for Fermentation Industry, Sunchang 56048, Republic of Korea; 3 Department of Statistics, Jeonbuk National University, Jeonju 54896, Republic of Korea; 4 Institute of Applied Statistics, Jeonbuk National University, Jeonju 54896, Republic of Korea; 5 Division of Food and Nutrition and Human Ecology Research Institute, Chonnam National University, Gwangju 61186, Republic of Korea; 6 K-Food Research Center, Jeonbuk National University, Jeonju 54896, Republic of Korea

**Keywords:** *Cheonggukjang*, white adipose tissue, lipid metabolism, gut microbiota-derived short-chain fatty acids, G-protein-coupled receptor

## Abstract

*Cheonggukjang* (CGJ) exerts anti-obesity and lipid-lowering effects; however, its effects on gut microbiota-derived short-chain fatty acid (SCFA) production, adipose G protein-coupled receptor (GPCR) expression, and the mechanistic associations between them have not been investigated. Thus, the current study aimed to investigate these relationships in relation to lipid metabolism in white adipose tissue (WAT). Two distinct CGJ samples were administered to high-fat/high-cholesterol diet-induced (HCFD) obese mice. CGJ slightly lowered body weight gain and significantly improved dyslipidemia and hepatic lipid accumulation, also improving WAT lipid metabolism-related gene expression. Additionally, CGJ reversed gut microbiota dysbiosis, increased the abundance of genera associated with SCFA production, and increased colonic SCFA levels. Adipose *Gpr41* and *Gpr109a* expressions were upregulated, and their mRNA levels were strongly correlated with systemic lipid indicators and WAT lipid-metabolism genes. To the best of our knowledge, this is the first study to provide evidence that CGJ modulates gut microbiota-derived SCFA levels and elevates adipose GPCR gene expression, suggesting that CGJ may exert anti-obesity and lipid-metabolism-alleviating effects through the gut microbiota–SCFA–adipose GPCR–WAT lipid metabolism axis.

## 1. Introduction

Obesity is one of the global health concerns, as it significantly increases the risk of various other metabolic disorders, including type 2 diabetes (T2DM) and cardiovascular diseases (CVDs). Obesity is primarily driven by a persistent imbalance between energy intake and expenditure, leading to the accumulation of excess energy as fat in white adipose tissues (WATs) [1,2]. Indeed, the frequent consumption of a Western diet, characterized by high-fat and high-cholesterol (HCFD), accelerates and exacerbates dysregulation of lipid metabolism, ultimately promoting WAT accumulation and dyslipidemia [3,4]. Lipid metabolism is coordinated by the network of transcription factors and/or enzymes. Sterol-responsive element binding protein 1c (SREBP1c), fatty acid synthase (FAS), and acetyl-CoA carboxylase (ACC) play major roles in adipogenesis and lipogenesis; on the other hand, catabolic metabolism is modulated by adipose triglyceride lipase (ATGL), hormone-sensitive lipase (HSL), and carnitine palmitoyltransferase1A (CPT1α) [1,2,5]. Recently, accumulating evidence suggested that lipid metabolism is closely linked to individuals’ gut environments and resident microbial communities, beyond the role of these key metabolic regulators.

The gut microbiota plays a pivotal role in host energy metabolism; accordingly, metabolites derived from it have been strongly implicated in multiple metabolic diseases, including obesity [6,7,8]. Among the diverse gut microbiota-derived metabolites, short-chain fatty acids (SCFA), including acetate (ACT, C2), propionate (PRO, C3), and butyrate (BUT, C4), act more as signaling molecules than as simple energy sources [9,10]. SCFAs activate G protein-coupled receptors (GPCRs), GPR41, GPR43, and GPR109A, which are expressed in diverse metabolic tissues, such as WATs, and modulate lipid metabolism [8,11]. For example, SCFA-mediated activation of adipose GPR43 decreases fat accumulation and promotes lipid utilization, suggesting a direct role of the gut microbiota-generated SCFA-GPCR axis in WATs and obesity-related lipid metabolism [11]. Although numerous studies have demonstrated that certain dietary interventions improve obesity by altering SCFA production [12,13], it remains unclear whether these interventions also affect GPCR expression in WATs and how alterations of receptor expression contribute to WAT lipid metabolism via the SCFA-GPCR axis.

As one of the representative Korean traditional fermented foods (KTFFs), *Cheonggukjang* (CGJ) has received significant attention for its diverse health-promoting effects, including anti-obesity and hypocholesterolemia effects [14,15,16,17,18]. For instance, CGJ markedly reduces body weight gain and WAT weight [14,15]; furthermore, it significantly reduces the expression of genes involved in adipogenesis in WATs, thereby alleviating obesity [18]. A recent study demonstrated that CGJ improves cholesterol and bile acid metabolism by restoring gut microbiota dysbiosis [14]. Additionally, CGJ consumption has significant alleviating effects on colonic diseases [19,20], such as constipation, implying its marked potential to restore disease-induced imbalances in the gut microbiota. However, CGJ’s effects on SCFA production and adipose GPCR expression, and the mechanistic relationships between these changes and its anti-obesity effects, have not been investigated.

Therefore, the present study aimed to elucidate the mechanisms underlying the alleviated effects of CGJ in lipid dysmetabolism by: (1) characterizing CGJ-induced changes in WAT lipid metabolism, including the expression of key genes involved in adipogenesis, lipogenesis, and lipolysis; (2) determining whether CGJ modulates gut microbiota composition and production of gut microbiota-derived SCFA; (3) investigating the impact of CGJ on adipose GPCR expression; and (4) exploring the correlations among gut microbiota composition, SCFA levels, adipose GPCR expression, and WAT lipid metabolism.

## 2. Materials and Methods

### 2.1. The Preparation of CGJ Samples

*Cheonggukjang* (CGJ) samples were provided by the Microbial Institute for Fermentation Industry (Sunchang, Jeollabuk-do, Republic of Korea) and were used as received without prior selection based on their microbial or compositional characteristics. According to the official methods of the Korean Food Code (Ministry of Food and Drug Safety, 2021) by BioFoodLab Co., Ltd. (Seoul, Republic of Korea), the nutritional composition of two CGJ samples was analyzed. The assessment of free amino acids and soy isoflavones contents in CGJ samples was analyzed by an accredited testing laboratory (Korea Health Functional Food Institute, Seongnam, Republic of Korea). 

The microbial characteristics of two CGJ samples were analyzed using next-generation sequencing (NGS) at the Microbial Institute for Fermentation Industry (Sunchang, Jeollabuk-do, Republic of Korea). Briefly, DNA was extracted from CGJ samples using the DNeasy PowerFood Microbial Kit (QIAGEN, Hilden, Germany) according to the manufacturer’s instructions. The 16S rRNA gene library was prepared following the 16S Metagenomic Sequencing Library Preparation protocol provided by Illumina (Illumina, San Diego, CA, USA). Sequencing was performed on the Illumina MiSeq platform using a paired-end 2 × 301 cycle configuration.

### 2.2. Animal Study

All animal procedures were reviewed and approved by the Institutional Animal Care and Use Committee of Jeonbuk National University (Approval No. NON2024-078-001). Male ICR mice (4 weeks old) were purchased from DooYeol Biotech (Seoul, Republic of Korea) and maintained under controlled environmental conditions (23 ± 1 °C, 60 ± 5% relative humidity, and a 12 h light/dark cycle). Following a one-week acclimation period to HCFD diet and environment, the animals were housed four mice per cage and randomly assigned into four experimental groups: the control group (CON; 10% fat of total kcal, *n* = 10), the HCFD group (HCFD; 1% cholesterol + 45% fat of total kcal, *n* = 10), and the HCFD supplemented with 30% of two different traditional CGJ groups (TMC24-10 and TCC24-6, *n* = 8 each) [15]. The CON and HCFD groups were shared with our previously published study [14], whereas the present study investigated different CGJ groups. Data from the shared CON and HCFD groups were statistically reanalyzed together with data from the current CGJ groups for the comparisons presented in this study. No animals were excluded from the primary analyses, and sample analyses were performed in a blind manner. Experimental diets were provided as pellets prepared according to the composition described in Supplementary Figure S1. During the experimental period (12 weeks), all mice had free access to diet and water. Food intake was monitored three times a week, and body weight was measured weekly. For fecal collection, mice were individually housed for 24 h immediately before dissection, and samples were collected separately from each mouse and stored at −80 °C until analysis, with individual mice serving as the experimental units for microbiome analysis. After 12 weeks, serum and tissues were collected after anesthesia and stored at −80 °C until analyzed. Briefly, serum samples were obtained from blood collected via retro-orbital bleeding under anesthesia; the samples were allowed to clot at room temperature, then centrifuged (3000 rpm for 10 min at 4 °C).

### 2.3. Histological Evaluation

Tissues from the liver, subcutaneous fat, and epididymal fat were immersed in 10% formalin for fixation over 48 h. The subsequent process for hematoxylin and eosin (H&E) staining was performed by KP&T Company (Cheongju, Chungcheongbuk-do, Republic of Korea). The stained slides were then evaluated and imaged using the EVOS™ M7000 Imaging System (ThermoFisher, Waltham, MA, USA, AMF7000). Number of hepatic lipid droplets and adipocytes in WATs were counted within the field and then differences in lipid droplet and adipocyte numbers among the groups were noted.

### 2.4. Assessment of Lipid and Hepatic Marker Profiles

Serum levels of triglycerides (TG, Cat. AM157S), total cholesterol (TC, Cat. AM202), high-density lipoprotein cholesterol (HDL, Cat. AM203-K), serum concentration of aspartate aminotransferase (AST, Cat. AM103-K), and alanine aminotransferase (ALT, Cat. AM102-K) were purchased from Asan Pharmaceutical Co., Seoul, Republic of Korea. Very low-density lipoprotein (VLDL-C) and low-density lipoprotein cholesterol (LDL-C) were calculated using Friedewald’s formula (VLDL-C = TG/5 and LDL-C = TC − HDL-C − TG/5). Lastly, apolipoprotein B (ApoB) was assessed using an ELISA kit (Abcam, Cambridge, UK, Cat. ab230932) according to the company’s protocol.

### 2.5. NGS Analysis

Bacterial community analysis of fecal samples was performed using NGS. Shortly, Genomic DNA from bacterial samples was isolated using a Fast DNA Spin kit (MP Bio Laboratories, Santa Ana, CA, USA). Amplification of the bacterial 16S rRNA gene was carried out using the universal primer pair 27F (5′-GAGTTTGATCMTGGCTCAG-3′) and 518R (5′-WTTACCGCGGCTGCTGG-3′), covering the V1–V3 hypervariable regions. According to the earlier primer set [21], PCR was performed on 16S rDNA using the following cycling conditions: 94 °C (5 min), 94 °C (30 s), 60 °C (45 s), and 72 °C (90 s) × 10 cycles. 94 °C (30 s), 55 °C (45 s), and 72 °C (90 s) × 20 cycles. Moreover, α-diversity and taxonomic composition were assessed using the Ez-Taxon database(PKSSU version 4.0) was used as a database.

### 2.6. Colonic SCFAs Measurement

Cecal luminal contents were collected during dissection, stored at −80 °C, and subsequently analyzed for SCFA contents at the Korea Research Institute of Bio-Medical Science (Daejeon, Republic of Korea). Briefly, a mixture of 1N HCl and 0.025 mL of 0.1 M isobutanol (internal standard) was vortexed for 10 min, and fatty acids were extracted with diethyl ether. The supernatant was collected after centrifugation (4 °C, 12,000 rpm, 5 min), and 1 µL aliquot of the supernatant was injected into a GC-MS system (7890 GC, Agilent Technologies, Santa Clara, CA, USA) equipped with a DB-FATWAX Ultra Inert column (Agilent Technologies, Santa Clara, CA, USA).

### 2.7. Quantitation of Real-Time PCR (qRT-PCR) Analysis

Total RNA was extracted from liver, epididymal fat, and subcutaneous fat using Trizol reagent (Takara Korea, Seoul, Republic of Korea). cDNA was synthesized with PrimeScript RT Master Mix (Takara, Kyoto, Japan). Quantitative real-time PCR was conducted on a 7500 Real-Time PCR System (Applied Biosystems, Foster City, CA, USA) using SYBR Green PCR Master Mix (Biosystems, Woolston, Warrington, UK). Primers were diluted to 25 pmol/μL before use. Gene expression was normalized to GAPDH and analyzed using the 2^−ΔΔCt^ method.

### 2.8. Statistical Analysis

Data are presented as mean ± SEM. Statistical analysis was performed using SPSS 23.0 (SPSS Inc., Chicago, IL, USA). Comparisons across groups were performed using one-way analysis of variance (ANOVA) followed by Duncan’s Multiple Range Test. Differences were considered statistically significant at *p* < 0.05, and significance levels are indicated as * *p* < 0.05, ** *p* < 0.01, and *** *p* < 0.001. For multiple correlation analyses, *p*-values were adjusted using the false discovery rate (FDR) method, and the resulting FDR-adjusted q-values were used to determine statistical significance. Additionally, to evaluate the consistency and robustness of associations between variables, correlation analyses were conducted using both Spearman’s rank correlation and Pearson’s correlation coefficient. Spearman’s rank correlation analysis was conducted as a sensitivity analysis to assess the robustness of the results and correlation coefficients, and *p*-values were reported. Additionally, Pearson’s correlation coefficient was used to assess linear relationships between variables. For correlation analyses, data from the two CGJ-treated groups were pooled, and statistical outliers were excluded before analysis.

## 3. Results

### 3.1. Characteristics of CGJ Samples

**Figure 1:**
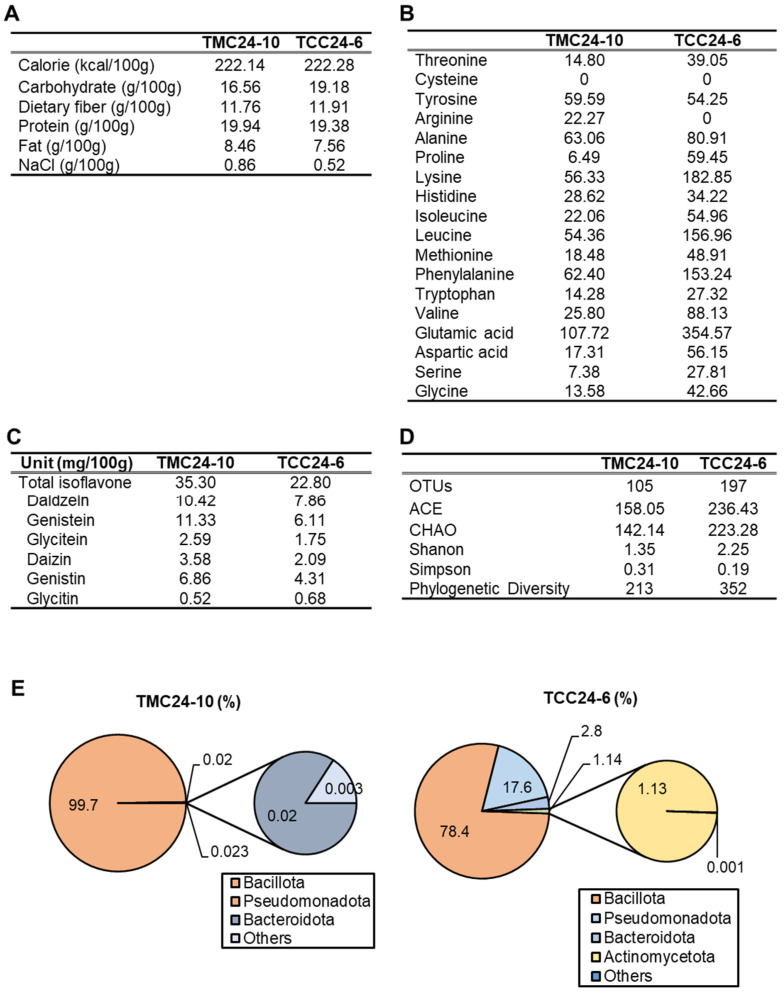
Characteristics of CGJs. (**A**) Nutritional composition, (**B**) composition of free amino acids, (**C**) contents of isoflavones, (**D**) α-diversity index, and (**E**) microbial composition of CGJs.

The nutritional composition of each CGJ sample is shown in Figure 1A.

Specifically, TMC24-10 (100 g) contained 16.56 g of carbohydrate, 11.76 g of dietary fiber, 19.94 g of protein, 8.46 g of fat, and 0.86 g of NaCl, while TCC24-6 (100 g) contained 19.18 g of carbohydrate, 11.91 g of dietary fiber, 19.38 g of protein, 7.56 g of fat, and 0.52 g of NaCl. In addition, the composition of free amino acids differed between the two CGJ samples (Figure 1B). The total isoflavone content was 35.30 mg/100 g for TMC24-10 and 22.80 mg/100 g for TCC24-6, respectively (Figure 1C).

The microbial characteristics of CGJ samples were analyzed using diversity and abundance metrics of microbial communities. Overall, most diversity and richness indicators were higher in TCC24-6 than in TMC24-10, whereas the Simpson index was lower in TCC24-6 (Figure 1D). The relative abundance of bacterial phyla in CGJ samples was also analyzed. The most abundant microbial community of TMC24-10 was Bacillota (99.7%), followed by Pseudomonadota (0.23%) and others (0.02%), while TCC24-6 was mainly dominated by Bacillota (78.4%), followed by Pseudomonadota (17.6%), Bacteroidota (2.8%), and others (1.13%). These findings suggest that the two CGJ samples have different nutritional, non-nutritional, and microbial characteristics.

### 3.2. CGJ Improves Systemic Lipid Profiles and WAT Metabolic Parameters in HCFD-Induced Obese Mice

**Figure 2:**
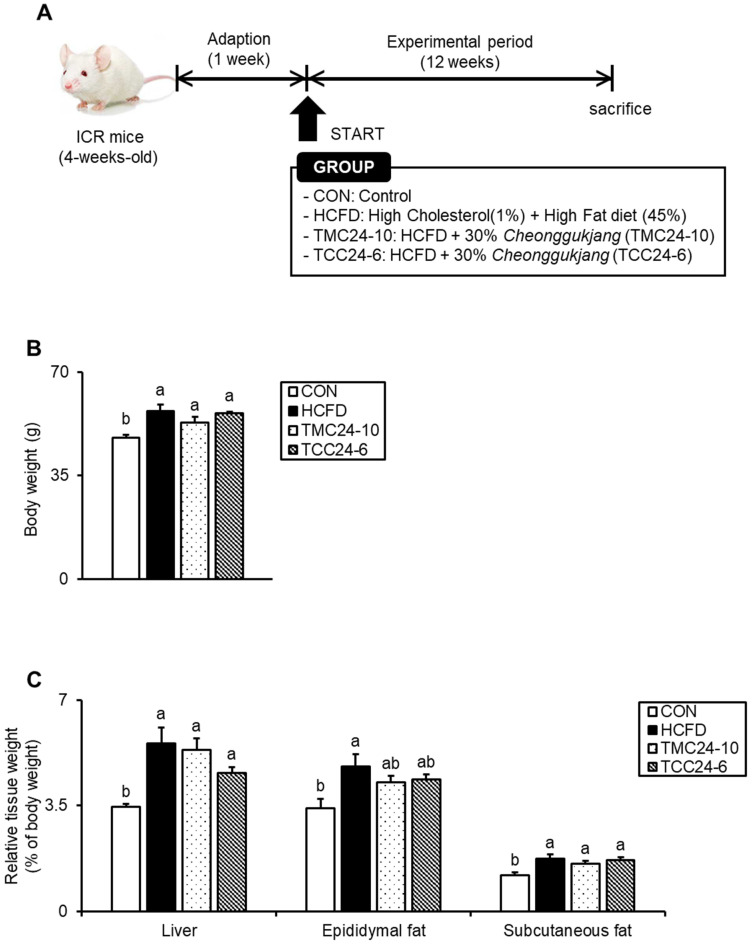
Effects of CGJ on systemic and metabolic parameters in HCFD-induced obese mice. (**A**) A schematic figure of experimental design, (**B**) body weight on the final day, and (**C**) relative weight of liver, epididymal fat, and subcutaneous fat. Values are mean ± SEM. Different letters (a, b) indicate differences among groups (ANOVA with Duncan’s test, *p* < 0.05; a > b).

The experimental design of this study is shown in Figure 2A. The effects of CGJ on body weight and serum lipid parameters were analyzed to confirm its anti-obesity and dyslipidemia-alleviating effects.

Feed efficiency was significantly higher in the TMC24-10 than in CON, whereas there were no significant differences among the other groups (Supplementary Figure S1B). In addition, energy efficiency was significantly higher in both CGJ groups compared with CON, while HCFD had no significant differences from the other groups (Supplementary Figure S1C). Compared to CON, HCFD had markedly increased body weight on the final day, whereas both TMC24-10 and TCC24-6 showed a slightly reduced tendency of body weight relative to HCFD (Figure 2B and Supplementary Figure S1D,E). Relative liver weight was significantly higher in all HCFD-fed groups compared to CON, while both CGJ groups showed a slight decrease relative to HCFD, without statistical significance (Figure 2C). Compared with CON, HCFD showed significantly increased relative epididymal fat and subcutaneous fat weight, whereas both CGJ groups had a lower tendency than HCFD without statistical significance (Figure 2C).

**Figure 3:**
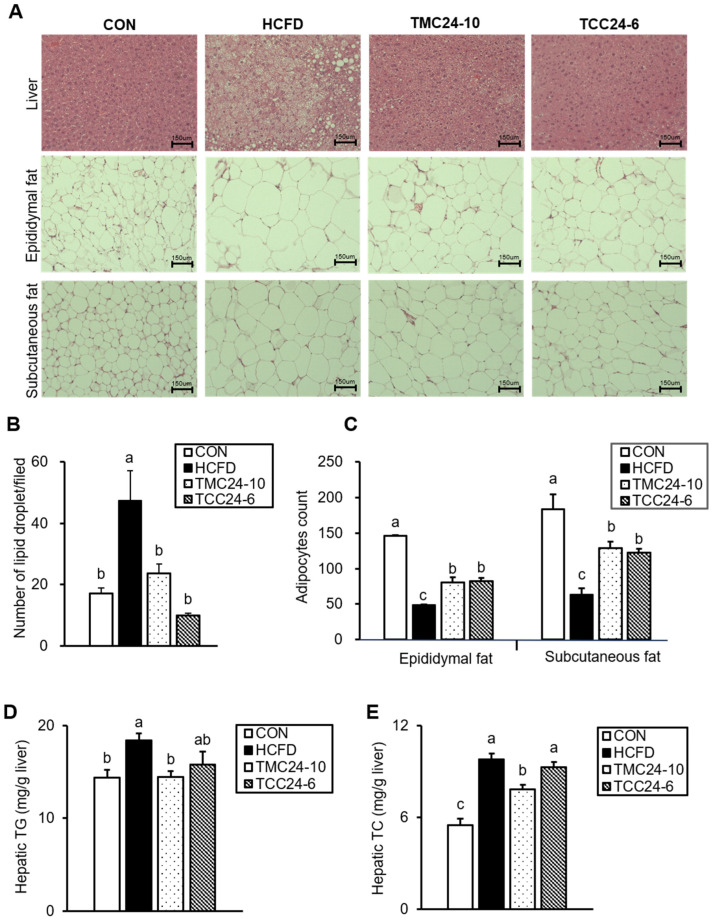
Effects of CGJ on fat accumulation in the liver and WATs of HCFD-induced obese mice. (**A**) Histological images of liver, subcutaneous fat, and epididymal fat (H&E staining, magnification 200×). Scale bar: 150 μm, (**B**) number of hepatic lipid droplet to area, (**C**) number of adipocytes to area in epididymal fat and subcutaneous fat, (**D**) hepatic TG level, and (**E**) hepatic TC level. Values are mean ± SEM. Different letters (a–c) indicate differences among groups (ANOVA with Duncan’s test, *p* < 0.05; a > c).

In addition to relative tissue weights, histological analysis of each tissue was performed. In the liver, HCFD showed a noticeably increased number of lipid droplets relative to CON, while both TMC24-10 and TCC24-6 showed significantly reduced hepatic lipid droplets compared with HCFD (Figure 3A,B). In the epididymal fat and subcutaneous fat tissues, HCFD had markedly enlarged adipocytes and increased numbers of adipocytes relative to CON, whereas both TMC24-10 and TCC24-6 showed decreased adipocyte size and lowered number of adipocytes compared with HCFD (Figure 3A,C). Consistent with the hepatic histological results, the levels of hepatic TG and TC were markedly elevated in HCFD relative to CON, whereas TMC24-10 showed a significant reduction in these levels compared with HCFD, and TCC24-6 showed a trend toward reduction without statistical significance (Figure 3D,E).

Next, the effects of CGJ on dyslipidemia were evaluated. Relative to CON, HCFD had a slight elevation in serum TG level, whereas both CGJ groups showed a lower trend than HCFD, with or without statistical significance (Table 1).

Serum TC levels in HCFD were significantly increased compared with CON, while both CGJ groups showed a marked reduction relative to HCFD. Compared with CON, HCFD had a slight elevation in VLDL-C levels, whereas both TMC24-10 and TCC24-6 showed a decreasing trend relative to HCFD, though the differences did not reach statistical significance. The LDL-C level in HCFD was markedly increased compared with CON, whereas TMC24-10 showed a slight decrease, and TCC24-6 showed a significant reduction compared with HCFD (Table 1). HCFD had a mild elevation in HDL-C levels compared to CON, while both TMC24-10 and TCC24-6 showed a significant upregulation of HDL-C levels compared to CON and HCFD. The level of ApoB in HCFD was also significantly increased relative to CON, whereas both CGJ groups showed a marked reduction compared to HCFD. Lastly, serum AST and ALT levels were measured to investigate hepatic damage. Compared to CON, all HCFD-fed groups had significantly higher AST levels, while both TMC24-10 and TCC24-6 showed a slight reduction compared to HCFD. The ALT levels in HCFD were significantly higher than in CON, while both TMC24-10 and TCC24-6 showed notably lower ALT levels compared to HCFD. Taken together, these observations indicate that CGJ alleviates body weight gain, lipid accumulation in the liver and WATs, and dyslipidemia, resulting in systemic improvement in HCFD-induced obese mice.

### 3.3. CGJ Restores Gut Microbial Diversity and Composition in HCFD-Induced Obese Mice

To investigate how CGJ modulates the gut microbial environment, the diversity and abundance of the intestinal microbiota were analyzed. In the analysis of α-diversity indices, including OTUs, ACE, Chao1, Shannon, and phylogenetic diversity, HCFD showed a significant decrease in diversity compared to CON, while this lower diversity was markedly restored in both CGJ groups (Table 2).

Additionally, β-diversity was analyzed using Bray–Curtis distance-based principal coordinate analysis (PCoA) to investigate differences in microbial community structure among groups (Supplementary Figure S2A). Bray–Curtis PCoA visually indicated that the TMC24-10 and TCC24-6 groups exhibited microbial community structures closer to CON than to HCFD. PERMANOVA further confirmed significant differences in microbial community composition among the four groups (*p* < 0.001; 999 permutations), and all pairwise comparisons remained significant after FDR correction (q < 0.001; Supplementary Figure S2A). At the phylum level, Firmicutes and Bacteroidetes were the most abundant in all groups (Supplementary Figure S2B). Relative to CON, HCFD showed increased Firmicutes and reduced Bacteroidetes levels, whereas both TMC24-10 and TCC24-6 reversed these trends compared with HCFD. At the genus level, *Staphylococcus* was the predominant genus across all groups (Supplementary Figure S3C). Compared with the HCFD, both CGJ groups exhibited greater similarities in microbial composition than CON, although no marked differences in dominant genera were observed. In summary, these observations imply that CGJ improves gut microbiota diversity and the structure of gut communities in HCFD-induced obese mice.

### 3.4. CGJ Increases the Abundance of Genera Associated with SCFA Production and Colonic SCFA Levels in HCFD-Induced Obese Mice

Obesity strongly leads to gut microbiota dysbiosis and alterations of gut microbiota-derived SCFA production [6,7]. As CGJ significantly restored the obesity-induced imbalance in the gut microbiota (Table 2 and Supplementary Figure S2), the genera associated with SCFA production and colonic SCFA levels were further investigated.

**Figure 4:**
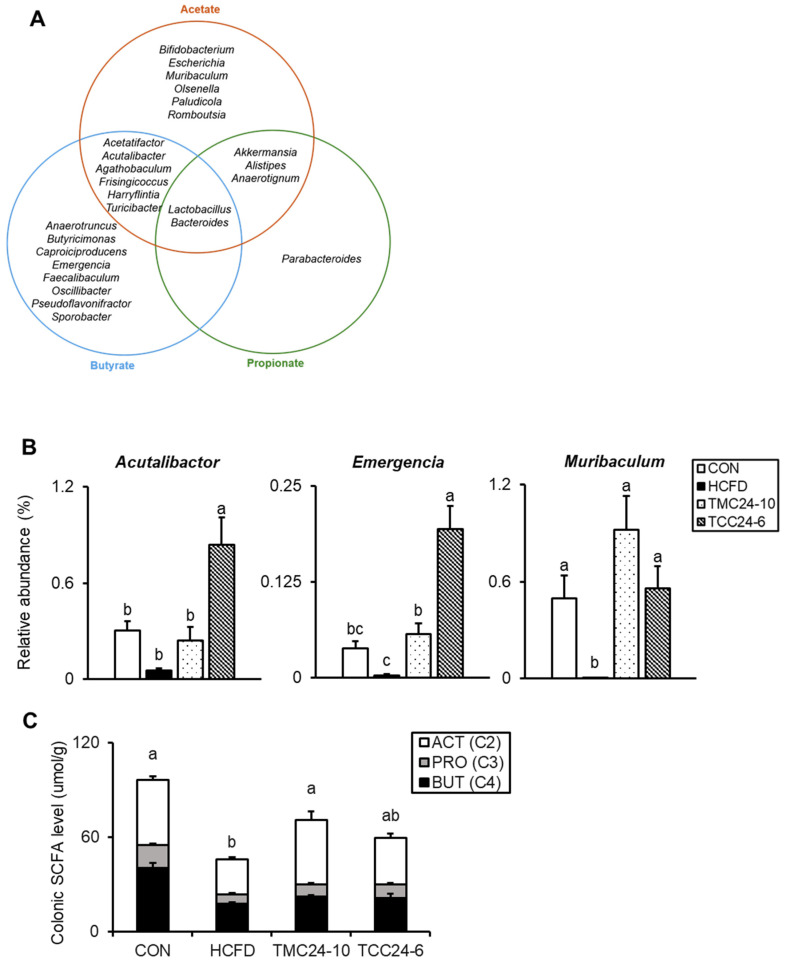
Effects of CGJ on the genera associated with SCFA production and colonic SCFA levels in HCFD-induced obese mice. (**A**) A Venn diagram of genera associated with SCFA production, (**B**) relative abundance of specific genera associated with SCFA production, and (**C**) colonic SCFA levels. Values are mean ± SEM. Different letters (a–c) indicate differences among groups (ANOVA with Duncan’s test, *p* < 0.05; a > c).

According to 16S rRNA gene metagenomic sequencing, genera previously associated with SCFA production were identified based on earlier studies [22,23,24] (Figure 4A). Although there was no statistical significance, *Acutalibacter* levels in HCFD were lower than in CON, while TMC24-10 showed an increasing trend compared to HCFD without statistical significance, and TCC24-6 had markedly elevated levels (Figure 4B). The levels of *Emergencia* in HCFD tended to be lower than in CON, while both TMC24-10 and TCC24-6 showed significantly higher levels than HCFD. HCFD showed strongly lowered levels of *Muribaculum* relative to CON, whereas both CGJ groups had significantly elevated levels compared with HCFD (Figure 4B). The levels of *Frisingicoccus* in both TMC24-10 and TCC24-6 were slightly higher than HCFD, although there was no statistical significance among all groups (Supplementary Figure S3A). The levels of *Harryflintia* in TMC24-10 tended to be higher than HCFD, and TCC24-6 showed a significant elevation of those levels, although there was no statistically significant difference between CON and HCFD. Lastly, both TMC24-10 and TCC24-6 showed a marked elevation in *Parabacteroides* levels compared with CON and HCFD (Supplementary Figure S3A).

Based on the significant alterations genera associated with SCFA production among the groups (Figure 4B and Supplementary Figure S3A), colonic SCFA levels were measured. Compared with CON, HCFD showed an overall reduction in SCFA levels, while TMC24-10 showed a significant elevation and TCC24-6 showed an increasing trend relative to HCFD (Figure 4C). Collectively, these findings suggest that CGJ modulates the abundance of genera associated with SCFA production and changes SCFA levels in HCFD-induced obese mice.

### 3.5. CGJ Modulates Adipose GPCR mRNA Expression and Gene Levels Associated with WAT Lipid Metabolism in HCFD-Induced Obese Mice

**Figure 5:**
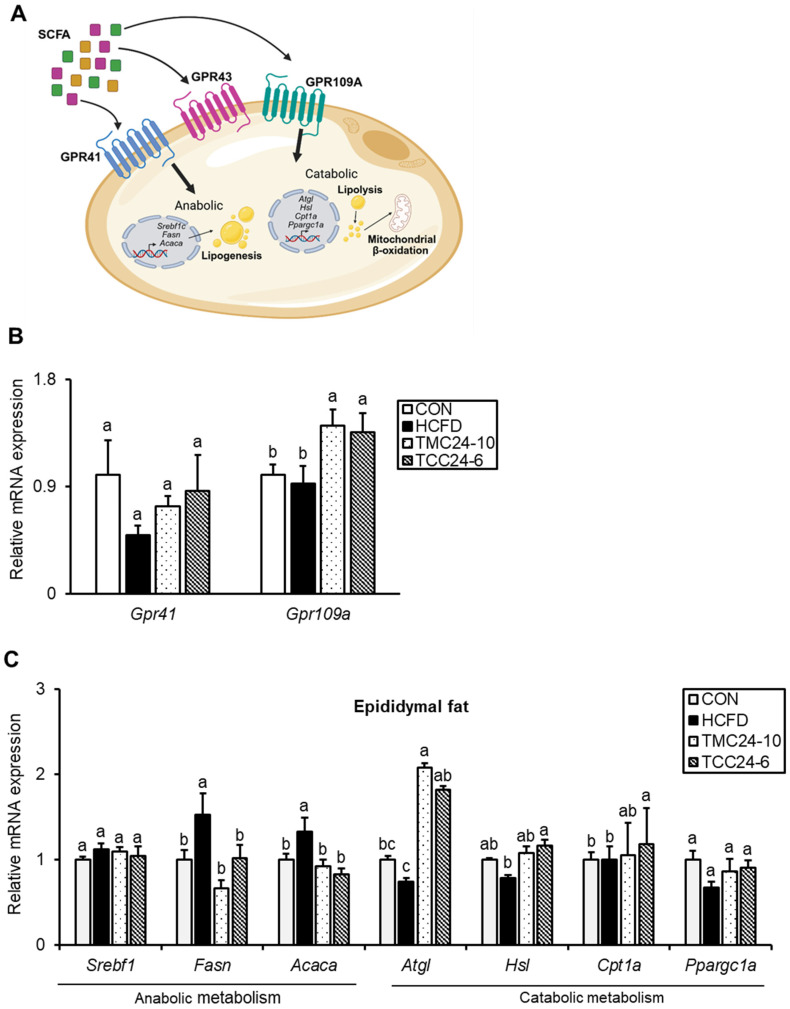
Effects of CGJ on adipose GPCRs gene expression and WAT lipid metabolism-related gene levels in epididymal fat of HCFD-induced obese mice. (**A**) A summary figure of the SCFA-GPCR axis in WATs, (**B**) relative mRNA expression of Gpr41 and Gpr109a in epididymal fat, and (**C**) relative mRNA expression of Srebf1, Fasn, Acaca, Atgl, Hsl, Cpt1a, and Ppargc1a in the epididymal fat. Values are mean ± SEM. Different letters (a–c) indicate differences among groups (ANOVA with Duncan’s test, *p* < 0.05; a > c). Different colors indicate individual SCFAs, including ACT, PRO, and BUT.

By activating adipose GPCRs, SCFA modulates lipid metabolism in WATs, including lipogenesis, lipolysis, and fatty acid oxidation [8,11] (Figure 5A); thus, the effects of CGJ on adipose GPCR expression and on genes involved in anabolic and catabolic lipid metabolism in WATs were analyzed.

Although there were no statistically significant differences among groups, the adipose *Gpr41* level in HCFD tended to be downregulated relative to CON, whereas both TMC24-10 and TCC24-6 showed a trend toward elevation (Figure 5B). Additionally, both CGJ groups showed a significant upregulation of adipose *Gpr109a* level relative to CON and HCFD, even though there was no difference between CON and HCFD. Additionally, there were no differences in adipose *Gpr43* levels among groups (Supplementary Figure S3B).

In epididymal fat, the levels of *Srebf1* did not differ among all groups (Figure 5C). *Fasn* and *Acaca* levels were significantly upregulated in HCFD relative to CON, whereas both CGJ groups showed a marked reduction compared with HCFD. HCFD showed a lower *Atgl* level than CON, whereas both TMC24-10 and TCC24-6 showed significant upregulation compared to HCFD. Compared to CON, HCFD showed a reduced trend in *Hsl* expression, and both CGJ groups showed upregulation relative to HCFD, with or without statistical significance (Figure 5C). There was no difference in *Cpt1a* expression between CON and HCFD, while both CGJ groups showed an elevated trend compared to CON and HCFD, with or without statistical significance. The levels of *Ppargc1a* in HCFD were slightly downregulated compared to CON, and both TMC24-10 and TCC24-6 showed an upward trend relative to HCFD, although no statistically significant differences were observed among all groups (Figure 5C). In subcutaneous fat, compared with CON, HCFD increased *Srebf1* expression and decreased gene expression involved in catabolic metabolism, *Hsl*, *Cpt1a*, and *Ppargc1a*; however, both TCC24-10 and TCC24-6 generally attenuated these HCFD-induced alterations (Supplementary Figure S3C).

### 3.6. Association Between Adipose GPCR Expression and Lipid Metabolism Indicators in CGJ-Treated Mice

As CGJ elevated adipose *Gpr41* and *Gpr109a* levels and altered mRNA expressions associated with lipid metabolism in WATs (Figure 5B,C), correlation analysis was conducted within the CGJ-treated group to evaluate whether GPCR levels were associated with systemic lipid metabolism indicators and gene expressions related to lipid metabolism in epididymal fat.

**Figure 6:**
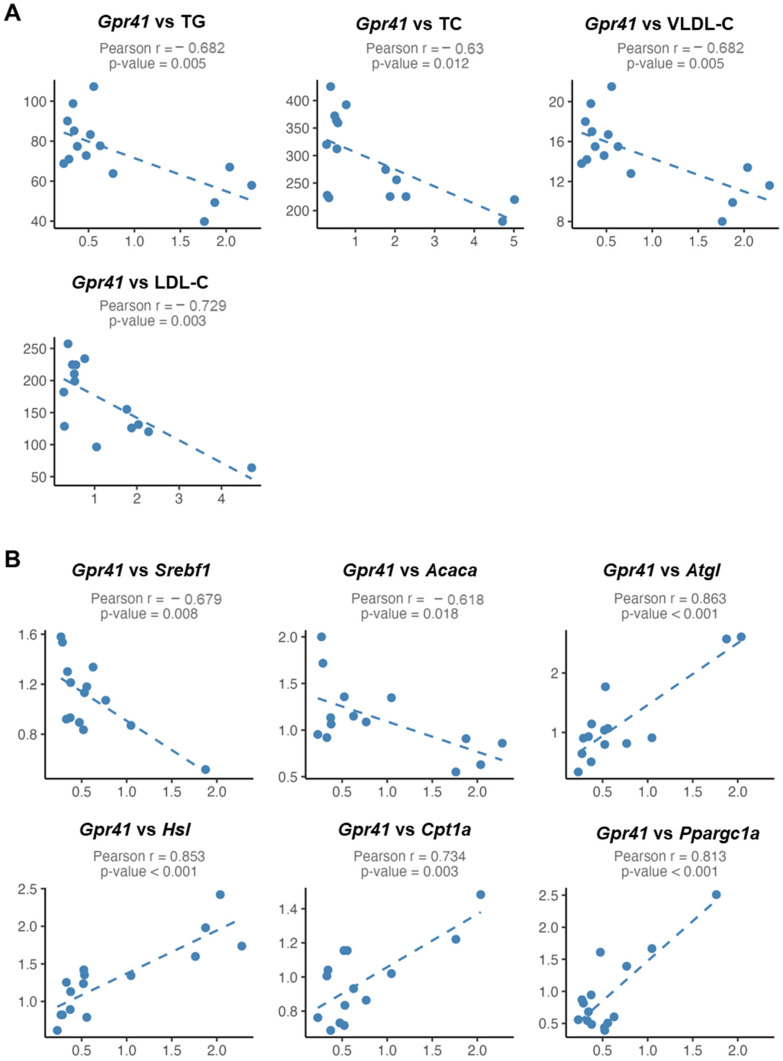
Association between epididymal fat Gpr41 expression and lipid metabolism indicators in the CGJ-treated group. Correlation analysis was performed within the CGJ-treated group (*n* = 14~15 mice per group). Gpr41 mRNA levels were analyzed alongside markers of (**A**) systemic lipid parameters and (**B**) anabolic and catabolic metabolism-related genes. The X-axis represents each Gpr41 mRNA expression, and the Y-axis represents lipid metabolism indicators or the related gene expression. Pearson’s correlation coefficients, r-values, and corresponding *p*-values are indicated in each panel.

Expression of adipose *Gpr41* was markedly correlated with systemic lipid metabolism indicators (Figure 6A and Supplementary Figure S4A).

The adipose mRNA level of *Gpr41* was inversely correlated with serum TG level (r = −0.682, *p*-value = 0.005), TC level (r = −0.63, *p*-value = 0.012), VLDL-C level (r = −0.682, *p*-value = 0.005), and LDL-C level (r = −0.729, *p*-value = 0.003). Moreover, adipose *Gpr41* expression was negatively correlated with hepatic lipid levels of TG and TC, although the correlation was not statistically significant (Supplementary Figure S4A). Adipose *Gpr41* was negatively correlated with gene levels associated with anabolic lipid metabolism, *Srebf1* (r = −0.679, *p*-value = 0.008) and *Acaca* (r = −0.618, *p*-value = 0.018) (Figure 6B). In contrast, adipose *Gpr41* was positively associated with gene expressions involved in catabolic lipid metabolism, *Atgl* (r = 0.863, *p*-value < 0.001), *Hsl* (r = 0.853, *p*-value < 0.001), *Cpt1a* (r = 0.734, *p*-value = 0.003), and *Ppargc1a* (r = 0.813, *p*-value < 0.001) (Figure 6B).

**Figure 7:**
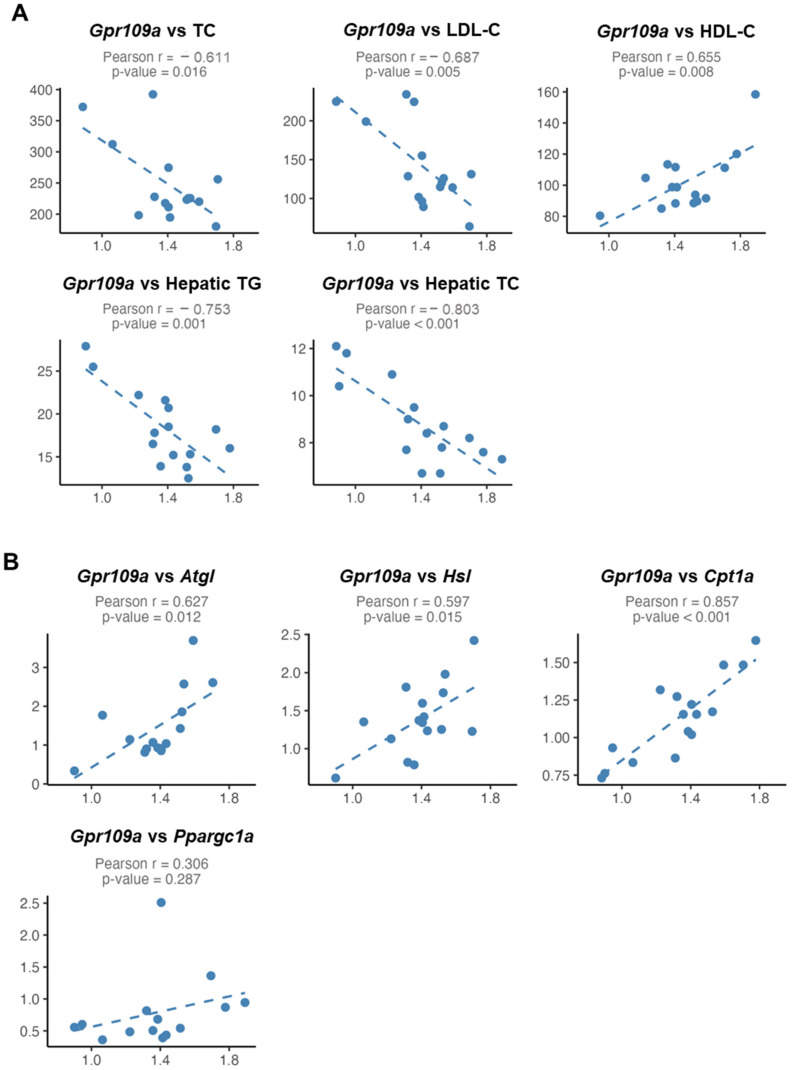
Association between epididymal fat *Gpr109a* expression and lipid metabolism indicators in the CGJ-treated group. Correlation analysis was performed within the CGJ-treated group (*n* = 14~15 mice per group). *Gpr109a* mRNA levels were analyzed alongside markers of (**A**) systemic lipid parameters and (**B**) anabolic and catabolic metabolism-related genes. The X-axis represents each *Gpr109a* mRNA expression, and the Y-axis represents lipid metabolism indicators or the related gene expression. Pearson’s correlation coefficients, r-values, and corresponding *p*-values are indicated in each panel.

The levels of *Gpr109a* were also strongly correlated with systemic lipid metabolism parameters (Figure 7A and Supplementary Figure S4B).

The adipose mRNA expression of *Gpr109a* was negatively correlated with serum TC level (r = −0.611, *p*-value = 0.016) and LDL-C level (r = −0.687, *p*-value = 0.005) but positively correlated with serum HDL-C level (r = 0.655, *p*-value = 0.008). Adipose *Gpr109a* expression was also inversely correlated with the levels of hepatic TG (r = −0.753, *p*-value = 0.001) and TC (r = −0.803, *p*-value < 0.001). Additionally, adipose *Gpr109a* levels were negatively correlated with serum TG level (r = −0.34, *p*-value = 0.214) and VLDL-C level (r = −0.428, *p*-value = 0.127), although the correlations were not statistically significant (Supplementary Figure S4B). Moreover, adipose *Gpr109a* expression was inversely correlated with gene expression of *Srebf1* (r = −0.254, *p*-value = 0.36) and *Acaca* (r = −0.44, *p*-value = 0.101), which are involved in anabolic lipid metabolism. without reaching statistical significance (Supplementary Figure S4B). In contrast, adipose *Gpr109a* was positively correlated with gene levels associated with catabolic lipid metabolism, *Atgl* (r = 0.627, *p*-value = 0.012), *Hsl* (r = 0.597, *p*-value = 0.015), *Cpt1a* (r = 0.857, *p*-value < 0.001), and *Ppargc1a* (r = 0.306, *p*-value = 0.287) (Figure 7B). A summary of the results from correlation analysis is shown in Supplementary Figure S4C. Taken together, these results indicate that higher adipose GPCR expression is strongly associated with improved lipid metabolism in CGJ-treated mice.

### 3.7. Heatmap of Spearman Correlation Coefficients Between Key Gut Microbiota, WATs Weight, Systemic Lipid Metabolism Indicators, Adipose GPCR, and Gene Levels Involved in WAT Lipid Metabolism

**Figure 8:**
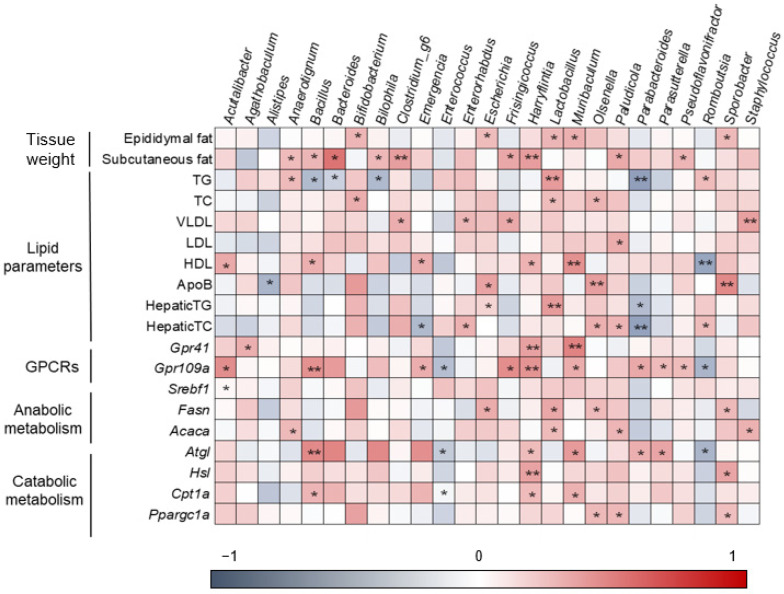
Heatmap of Spearman correlation coefficients between key gut microbiota, tissue weight, lipid metabolism indicators, adipose GPCRs gene, and genes related to WAT lipid metabolism. The intensity of the color represents the degree of association (blue, negative correlation; red, positive correlation). Statistical significance was determined using FDR-adjusted *q*-values * (*q* < 0.05) and ** (*q* < 0.01).

To examine the relationships among the gut microbiota, lipid metabolism indicators, and adipose GPCR expression, Spearman’s correlation analysis was performed (Figure 8). The levels of *Bifidobacterium*, *Escherichia*, *Lactobacillus*, *Muribaculum*, *and Sporobacter* were positively correlated with epididymal fat weight, while *Bacteroides*, *Clostridium_g6*, and *Harryflintia* were strongly correlated with subcutaneous fat weight.

Distinct microorganisms were directly associated with the systemic lipid metabolism index. With serum TG level, *Bacillus*, *Bacteroides*, *Bilophila*, and *Parabacteroides* were negatively correlated, whereas *Anaerotignum*, *Lactobacillus*, and *Romboutsia* were positively correlated. *Acutalibacter*, *Bacillus*, *Emergencia*, *Harryflintia*, and *Muribaculum* were directly correlated with serum HDL-C level, while *Romboutsia* was inversely correlated with serum HDL-C level. Hepatic TC level was inversely correlated with *Emergencia* and *Parabacteroides*, while *Enterorhabdus*, *Olsenella*, *Paludicola*, and *Romboutsia* were directly associated with hepatic TC contents.

Diverse microorganisms were also markedly associated with adipose GPCR gene expression and gene levels involved in lipid metabolism in epididymal fat. *Agathobaculum*, *Harryflintia*, and *Muribaculum* were positively correlated with adipose *Gpr41* expression. With adipose *Gpr109a* expression, *Acutalibacter*, *Bacillus*, *Emergencia*, *Frisingicoccus*, *Harryflintia*, and *Muribaculum* were positively correlated, while *Enterococcus* and *Romboutsia* were inversely correlated with adipose *Gpr109a* level. *Escherichia*, *Lactobacillus*, and *Olsenella* were positively correlated with *Fasn* gene expression. *Bacillus*, *Harryflintia*, *Muribaculum*, and *Sporobacter* were positively correlated with catabolic metabolism-associated gene expression, including *Atgl*, *Hsl*, *Cpt1a*, and *Ppargc1a*, while *Enterococcus* and *Romboutsia* were negatively correlated with *Atgl* and *Cpt1a* expression. In summary, these observations suggest complex associations between the gut microbiota and host parameters, including WAT weight, indicators of lipid metabolism, adipose GPCR expression, and lipid-metabolism-related gene expression in fat tissue.

## 4. Discussion

As a representative of KTFFs, CGJ elicits multiple health-promoting effects (e.g., anti-obesity and dyslipidemia-improving effects) by reducing WAT gain and improving lipid metabolism by regulating gene expression in the liver and WATs [14,16,17]; however, the possible mechanisms of the anti-obesity effects of CGJ, particularly its effects on gut microbiota composition, gut microbiota-derived SCFA levels, adipose GPCR expression, and their correlations, have not yet been investigated to date. The current study demonstrated that CGJ significantly improved systemic lipid indicators and lipid metabolism-related gene expression in WAT. Additionally, CGJ strongly restored gut microbiota, reduced dysbiosis, increased colonic SCFA levels, and upregulated adipose GPCR expression, with GPCR levels showing a marked correlation with systemic lipid profiles and WAT lipid-metabolism genes. Finally, the recovery of gut microbiota dysbiosis was associated with body weight, systemic lipid parameters, adipose gene levels involved in WAT lipid metabolism, and adipose GPCR mRNA levels.

The alleviative effects of CGJ in obesity and dyslipidemia have been well demonstrated [14,15,16,17,18]. In accordance with previous findings, the present study also found that CGJ intake ameliorates body weight gain, hepatic lipid accumulation, adipogenesis, and dyslipidemia by lowering serum TG, TC, and ApoB levels and increasing HDL-C levels. It has been reported that single or combined administration of soy isoflavones (e.g., genistein and daidzein) can strongly reduce body weight gain, visceral fat mass, and dyslipidemia, and impede adipogenesis [25,26]. Additionally, specific amino acid mixtures have been shown to improve energy metabolism, thereby lowering obesity risk [27,28]. In the present study, TMC24-10 had a higher total isoflavone content than TCC24-6, whereas TCC24-6 exhibited markedly higher levels of several free amino acids, including lysine, leucine, methionine, and glutamic acid, relative to TMC24-10. These compositional differences suggest that the modest discrepancies observed between the two CGJ samples in several systemic indicators (e.g., hepatic TG and TC levels) may, at least in part, reflect differences in bioactive compound content and amino acid profiles. Despite their distinct compositional profiles, both CGJ samples generally produced similar beneficial effects, suggesting that the metabolic effects of CGJ are relatively robust across compositions. Importantly, CGJ is a representative synbiotic food, providing both probiotic bacteria (e.g., Bacillus subtilis) and physiologically advantageous compounds (e.g., poly-γ-glutamic acid, γ-PGA) [29,30], thereby potentially regulating gut microbiota and host metabolism. Moreover, because non-fermented soybean control was not included in the present study, the effects specifically attributable to fermentation cannot be distinguished from those of the overall CGJ food matrix. Therefore, it will be important to examine in future studies whether the anti-obesity and dyslipidemia-ameliorative effects of CGJ observed in this study are primarily driven by fermentation-specific components and/or the complex synbiotic matrix or are attributable to specific amino acids and/or isoflavones.

The recovery roles of CGJ in disease-derived changes in gut microbiota composition and their correlation with disease improvements have been suggested [14,19,20]; however, the effects of CGJ on genera associated with SCFA production and colonic SCFA levels have not been investigated previously. Moreover, according to earlier studies, certain dietary interventions alter SCFA production [12,13]; however, the detailed molecular mechanisms by which these alterations translate into improvements in WAT lipid metabolism remain incompletely defined. The current study observed that CGJ not only strongly reversed obesity-induced gut community dysbiosis but also increased the abundance of genera associated with SCFA production and elevated colonic SCFA levels. Furthermore, CGJ upregulated adipose *Gpr41* and *Gpr109a* gene expression and improved WAT lipid metabolism-related gene expression, and these GPCR gene levels were markedly associated with systemic lipid indicators and both anabolic and catabolic lipid metabolism genes in WAT, suggesting an important association between increased adipose GPCR gene expression and CGJ-mediated anti-obesity effects and lipid-lowering effects. However, genus-level 16S rRNA sequencing cannot directly confirm SCFA-producing capacity; thus, future metagenomic and functional studies are needed to validate the roles of these genera in SCFA production. Furthermore, in future studies, in vitro GPCR knockdown models (e.g., via siRNA transfection) will be essential for understanding the GPCR-dependent molecular mechanisms, including signaling pathways (e.g., the AMPK pathway), that underlie CGJ’s effects on the SCFA–adipose GPCR axis.

Another representative KTFF, Gochujang, remodels gut microbiota composition, and specific microbiota are closely correlated with disease-related indicators, including colorectal cancer burden, hepatic inflammation, and constipation [31,32]. Consistent with earlier observations, this study demonstrated that CGJ-induced recovery of gut microbiota dysbiosis was strongly associated with body weight, systemic lipid profiles, adipose GPCR mRNA levels, and WAT lipid metabolism-related genes, supporting a potential association between the gut microbiota–SCFA–adipose GPCR axis and the anti-obesity and dyslipidemia-alleviating effects of CGJ. Although these associations do not demonstrate causality, they suggest that increased adipose GPCR expression may be associated with the anti-obesity and dyslipidemia-alleviating effects of CGJ and alterations in WAT lipid metabolism. In future studies, receptor-specific inhibitors or activators in preclinical models will be valuable for understanding the individual contributions of GPR41 and GPR109A to CGJ-mediated improvements in WAT lipid metabolism, as well as their anti-obesity and ameliorative effects on lipid dysmetabolism. Additionally, future research regarding interrogating the functional roles of specific bacteria and CGJ-responsive bacteria will be essential to clarify how distinct microbial communities modulate the gut microbiota–SCFA–adipose GPCR axis and CGJ’s health benefits.

## 5. Conclusions

To the best of our knowledge, the present study is the first to provide evidence that CGJ upregulates adipose GPCR expression and modulates colonic levels of gut microbiota-derived SCFAs, offering a novel perspective that certain dietary interventions may regulate tissue-specific GPCR levels in vivo. CGJ significantly ameliorated dyslipidemia and hepatic lipid accumulation while reversing obesity-induced gut microbiota dysbiosis and increasing the abundance of genera associated with SCFA production and colonic SCFA levels. Furthermore, adipose *Gpr41* and *Gpr109a* mRNA levels were strongly correlated with systemic lipid indicators and WAT lipid-metabolism genes, collectively suggesting that CGJ may exert its anti-obesity and lipid-lowering effects in association with an integrated gut microbiota–SCFA–adipose GPCR–WAT lipid metabolism axis. However, this study is notably limited in that all findings regarding adipose GPCR and WAT lipid metabolism-related targets rely solely on gene-level data; future studies should validate these findings at the protein level and employ in vitro GPCR knockdown models to further establish the causal molecular mechanisms underlying the CGJ–SCFA–adipose GPCR axis.

## Figures and Tables

**Table 1:** The levels of serum lipid indicators.

	CON	HCFD	TMC24-10	TCC24-6
TG (mg/dL)	81.6 ± 5.9 ^ab^	84.0 ± 9.7 ^a^	64.6 ±5.7 ^ab^	58.8 ± 6.6 ^b^
TC (mg/dL)	154.1 ± 5.7 ^d^	301.8 ± 18.5 ^a^	250.8 ± 17.9 ^b^	207.1 ± 6.3 ^c^
VLDL-C (mg/dL)	16.3 ± 1.2 ^ab^	18.2 ± 2.3 ^a^	13.7 ± 1.3 ^ab^	12.9 ± 1.3 ^b^
LDL-C (mg/dL)	62.6 ± 3.0 ^c^	142.5 ± 11.1 ^a^	136.5 ± 11.6 ^a^	96.1 ± 8.1 ^b^
HDL-C (mg/dL)	78.8 ± 2.6 ^b^	83.1 ± 9.1 ^b^	122.6 ± 6.5 ^a^	106.0 ± 4.6 ^a^
ApoB (mg/dL)	46.9 ± 6.6 ^ab^	66.9 ± 13.7 ^a^	29.8 ± 6.8 ^b^	33.1 ± 7.5 ^b^
AST (U/L)	57.3 ± 1.8 ^b^	71.8 ± 3.7 ^a^	60.2 ± 3.8 ^a^	56.3 ± 0.8 ^a^
ALT (U/L)	100.4 ± 1.2 ^c^	151.1 ± 4.8 ^a^	134.7 ± 5.2 ^b^	124.5 ± 3.1 ^b^

Data for the CON and HCFD groups, some of which were previously reported in [14], were reanalyzed for comparison with the CGJ groups. Values are mean ± SEM. Different letters (a–d) indicate differences among groups (ANOVA with Duncan’s test, *p* < 0.05; a > d).

**Table 2:** Gut microbial alpha-diversity indices.

	CON	HCFD	TMC24-10	TCC24-6
OTUs	481.00 ± 50.0 ^a^	330.21 ± 28.6 ^c^	506.83 ± 34.5 ^a^	380.73 ± 10.7 ^b^
ACE	550.90 ± 51.3 ^b^	414.89 ± 27.6 ^d^	593.78 ± 33.7 ^a^	468.05 ± 29.0 ^c^
CHAO	553.56 ± 44.7 ^a^	404.27 ± 20.0 ^c^	578.96 ± 41.3 ^a^	449.14 ± 22.5 ^b^
Shannon	3.77 ± 0.4 ^a^	3.10 ± 0.3 ^b^	3.94 ± 0.3 ^a^	3.98 ± 0.1 ^a^
Simpson	0.06 ± 0.0 ^b^	0.09 ± 0.0 ^a^	0.05 ± 0.0 ^bc^	0.04 ± 0.0 ^c^
Phylogenetic Diversity	699.32 ± 47.3 ^a^	516.09 ± 35.3 ^c^	721.36 ± 41.7 ^a^	580.05 ± 18.1 ^b^

Values are mean ± SEM. Different letters (a–d) indicate differences among groups (ANOVA with Duncan’s test, *p* < 0.05; a > d).

## Data Availability

All data are available upon reasonable request to the corresponding author.

## Supplementary Materials

The following supporting information can be downloaded at https://www.mdpi.com/article/10.3390/foods15152653/s1. Figure S1: Effects of CGJ on body weight; Figure S2: Effect of CGJ on gut microbial composition in HCFD-induced obese mice; Figure S3: Effects of CGJ on SCFA-producing bacteria, *Gpr43* gene expression in epididymal fat, and lipid metabolism-related gene levels in subcutaneous fat of HCFD-induced obese mice; Figure S4: Association between epididymal fat *Gpr41* and *Gpr109a* expression and lipid metabolism indicators in the CGJ-treated group.

## Abbreviations

The following abbreviations are used in this manuscript:

ACC	Acetyl-CoA carboxylase
ACT	Acetate
AMPK	AMP-activated protein kinase
ApoB	Apolipoprotein B
AST	Aspartate aminotransferase
ALT	Alanine aminotransferase
ATGL	Adipose triglyceride lipase
BUT	Butyrate
CGJ	*Cheonggukjang*
CPT1α (CPT1A)	Carnitine palmitoyl transferase 1 alpha
FAS	Fatty acid synthase
GPCR	G protein-coupled receptor
HCFD	High-fat/high-cholesterol diet
HDL-C	High-density lipoprotein cholesterol
HSL	Hormone-sensitive lipase
KTFF	Korean traditional fermented food
LDL-C	Low-density lipoprotein cholesterol
PRO	Propionate
SCFA	Short-chain fatty acid
SREBP1c	Sterol regulatory element-binding protein 1c
TC	Total cholesterol
TG	Triglyceride
VLDL-C	Very low-density lipoprotein cholesterol
WAT	White adipose tissue
