# Supplementary material for: Cheonggukjang Attenuates Lipid Dysmetabolism by Modulating Gut Microbiota-Derived SCFA Production and G-Protein-Coupled Receptor Levels in White Adipose Tissue
Source: Foods. 2026 Jul 28;15(15):2653. doi: 10.3390/foods15152653 (PMC13463837; doi:10.3390/foods15152653)
Supplement: Supplementary file 1 [file foods-15-02653-s001.zip › foods-4449284-supplementary.pdf]

Supplementary Figure S1. Effects of CGJ on body weight

(A) Experimental diet composition, **(B)** Feed efficiency, **(C)** Energy efficiency, **(D)** Body weight at the initial and final points of the experiments, and **(E)** A curve graph of body weight**.** Values are mean ± SEM. Different letters (a–c) indicate differences among groups (ANOVA with Duncan's test, $p < 0.05$; a > c).

Supplementary Figure S2. Effect of CGJ on gut microbial composition in HCFD-induced obese mice

(A) Principal coordinate analysis (PCoA) plots based on the Bray–Curtis dissimilarity index. Pairwise PERMANOVA was performed with 999 permutations, and q-values were adjusted for multiple comparisons using the false discovery rate (FDR) method. **(B)** Relative abundance of microbial communities at the phylum level, and **(C)** Relative abundance of microbial communities at the genus level.

Supplementary Figure S3. Effects of CGJ on SCFA-producing bacteria, *Gpr43* gene expression in epididymal fat, and lipid metabolism-related gene levels in subcutaneous fat of HCFD-induced obese mice

(A) Relative abundance of specific genera of SCFA-producing bacteria, which was elevated by GCJ, **(B)** Relative mRNA expression of *Gpr43* in epididymal fat, and **(C)** Relative mRNA expression levels of *Srebf1, Fasn, Acaca, Atgl, Hsl, Cpt1a, Ppargc1a,* and *Ucp1* in the subcutaneous fat. Values are mean ± SEM. Different letters (a–c) indicate differences among groups (ANOVA with Duncan's test, $p < 0.05$; a > c).

Supplementary Figure S4. Association between epididymal fat *Gpr41* and *Gpr109a* expression and lipid metabolism indicators in the CGJ-treated group

Correlation analysis was performed within the CGJ-treated group (n=14~15 mice per group). **(A)** *Gpr41* mRNA levels were analyzed alongside markers of systemic lipid parameters, **(B)** *Gpr109a* mRNA levels were analyzed alongside markers of systemic lipid parameters, anabolic and catabolic metabolism-related genes, and **(C)** Values and strength results of Pearson and Spearman correlations between adipose GPCRs and indicators. The X-axis represents each *Gpr41* or *Gpr109a* mRNA expression, and the Y-axis represents lipid metabolism indicators or the related gene expression. Pearson's correlation coefficients, R-values, and corresponding p-values are indicated in each panel.

Supplementary Figure S1. Lee et al.

A

Components (g)	CON	HCFD	TMC24-10	TCC24-6
**Casein**	200	195	134.57	136.27
**Methionine, DL**	3	3	3	3
**Sucrose**	550	350	350	350
**Lodex**	150	100	49.82	41.88
**Starch, Corn**	4	50	50	50
**Fiber**	50	50	50	50
**Butter, Anhydrous**	0	200	174.36	177.09
**Corn Oil**	0	10	10	10
**Soybean Oil**	25	0	0	0
**Lard**	20	0	0	0
**Mineral**	50	35	35	35
**Calcium Carbonate**	0	4	4	4
**Choline Bitartrate**	2	2	2	2
**Vitamin C**	1	1	1	1
**Ethoxyquin**	0	0.04	0.04	0.04
**Cholesterol, NF**	0	10	10	10
Cheonggukjang	0	0	262.14	261.08
**Total**	1055.05	1010.04	1135.93	1131.36

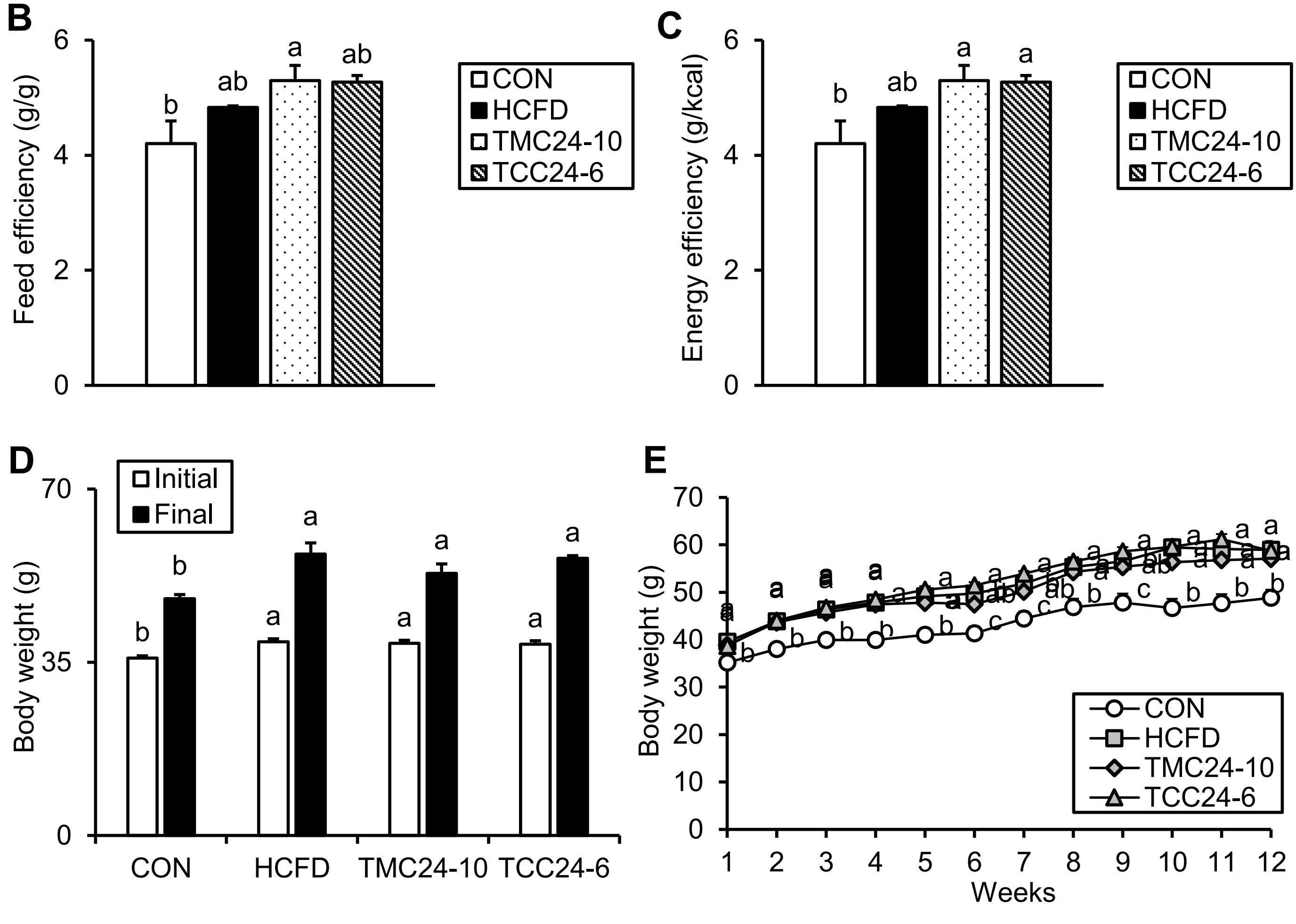

Supplementary Figure S2. Lee et al.

A

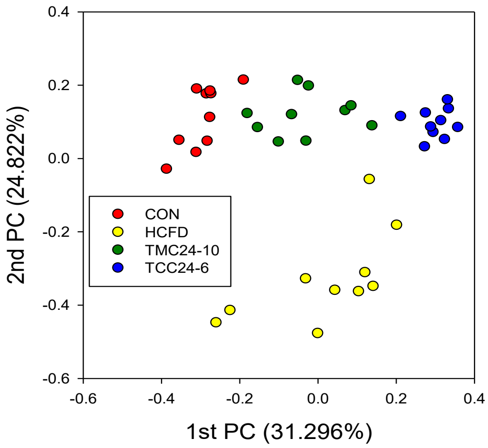

Comparison	pseudo-F	p-value	FDR-adjusted q-value
CON vs. HCFD	14.25	<0.001	<0.001
CON vs. TMC24-10	9.38	<0.001	<0.001
CON vs. TCC24-6	28.68	<0.001	<0.001
HCFD vs. TMC24-10	11.78	<0.001	<0.001
HCFD vs. TCC24-6	15.90	<0.001	<0.001
TMC24-10 vs. TCC24-6	14.96	<0.001	<0.001

B

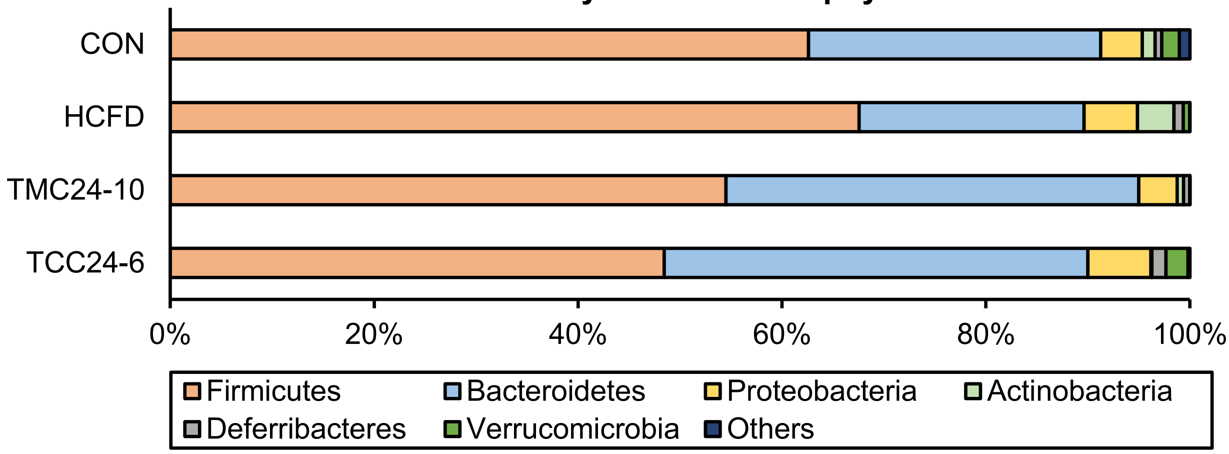

C

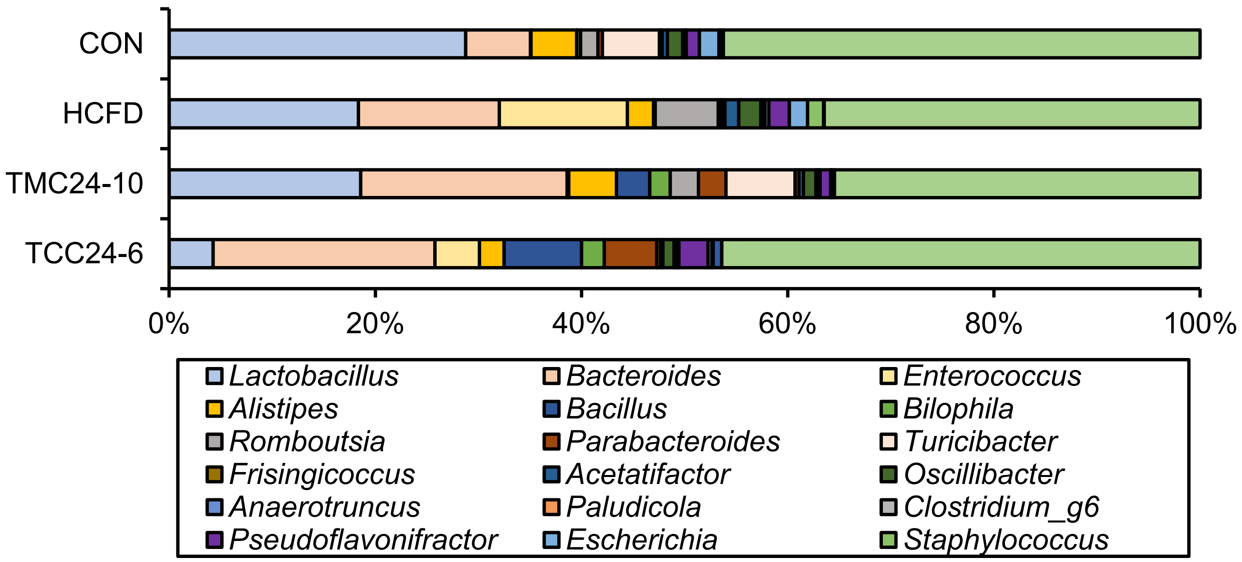

A

	CON	HCFD	TMC24-10	TCC24-6
Acutalibacter	0.304 ± 0.059^{b}	0.052 ± 0.016^{b}	0.243 ± 0.083^{b}	0.842 ± 0.168^{a}
Emergencia	0.039 ± 0.009^{bc}	0.003 ± 0.002^{c}	0.057 ± 0.014^{b}	0.194 ± 0.029^{a}
Frisingicoccus	0.263 ± 0.044^{a}	0.278 ± 0.051^{a}	0.350 ± 0.066^{a}	0.362 ± 0.059^{a}
Harryflintia	0.025 ± 0.007^{b}	0.023 ± 0.007^{b}	0.073 ± 0.019^{b}	0.231 ± 0.049^{a}
Muribaculum	0.495 ± 0.144^{a}	0.002 ± 0.001^{b}	0.921 ± 0.211^{a}	0.560 ± 0.134^{a}
Parabacteroides	0.439 ± 0.099^{c}	0.251 ± 0.058^{c}	2.582 ± 0.368^{b}	5.201 ± 0.618^{a}
Sporobacter	0.090 ± 0.016^{a}	0.044 ± 0.027^{a}	0.083 ± 0.016^{a}	0.050 ± 0.010^{a}

B

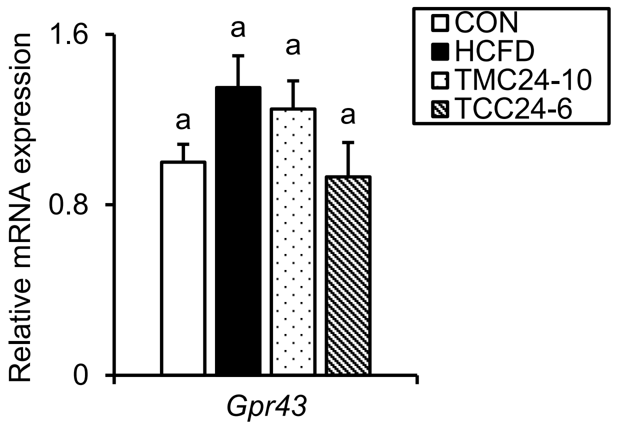

C

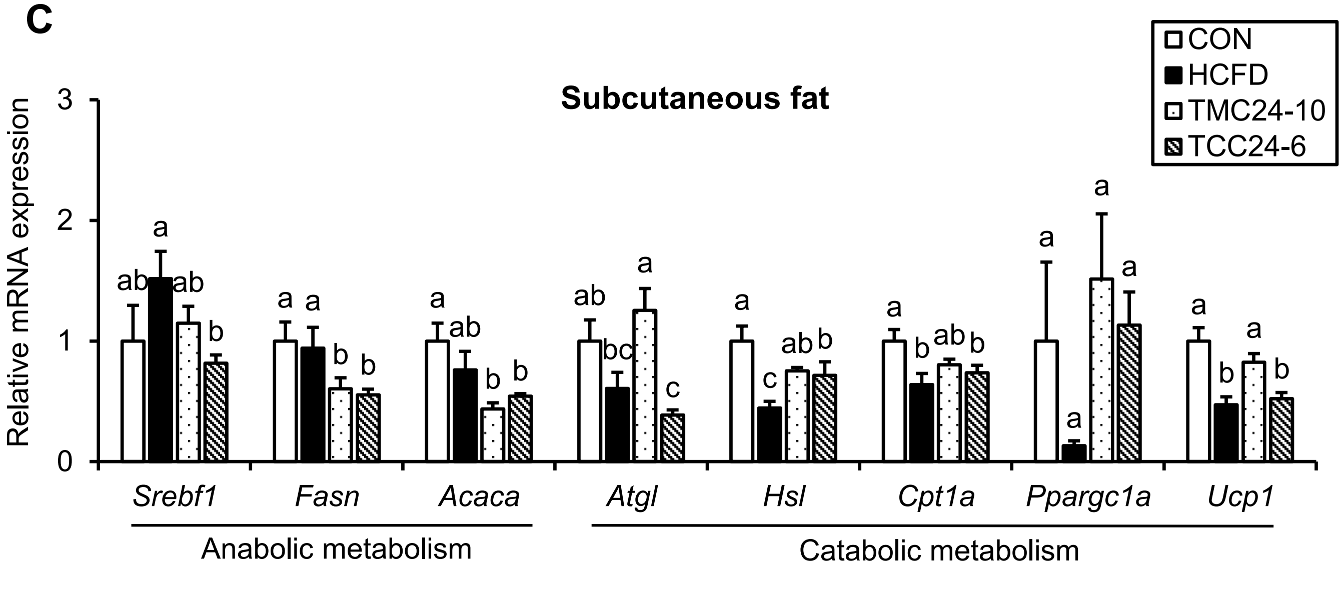

A

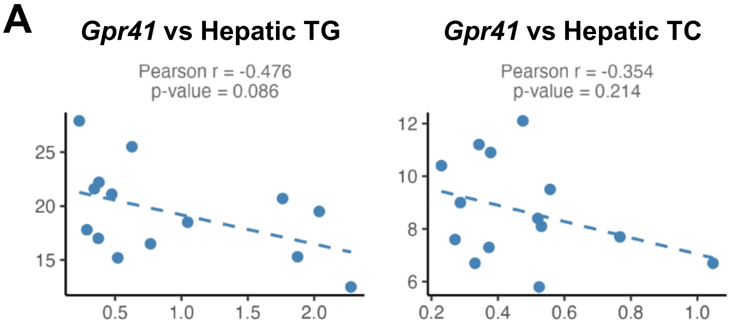

B

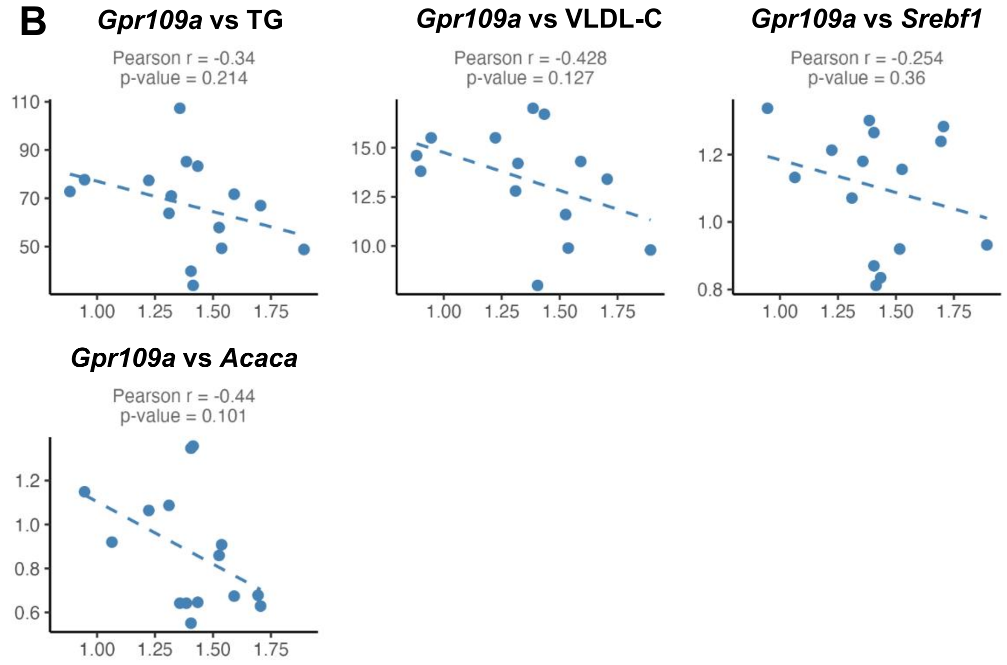

C

	Receptor	Indicators	Pearson r	p-value	Spearman ρ	p-value	Strength
**Lipid parameters**	*Gpr41*	TG	-0.682	0.005	-0.575	0.027	Strong
	Gpr41	TC	-0.630	0.012	-0.500	0.06	Moderate
	Gpr41	VLDL-C	-0.682	0.005	-0.583	0.023	Strong
	Gpr41	LDL-C	-0.729	0.003	-0.604	0.025	Strong
	Gpr109a	TC	-0.611	0.016	-0.393	0.147	Moderate
	Gpr109a	LDL-C	-0.687	0.005	-0.597	0.019	Strong
	Gpr109a	HDL-C	0.655	0.008	0.438	0.103	Strong
	Gpr109a	Hepatic TG	-0.753	0.001	-0.624	0.013	Strong
	Gpr109a	Hepatic TC	-0.803	<0.001	-0.715	0.003	Very Strong
**Anabolic metabolism**	*Gpr41*	*Srebf1*	-0.679	0.008	-0.543	0.048	Moderate
	Gpr41	*Acaca*	-0.618	0.018	-0.591	0.029	Moderate
**Catabolic metabolism**	*Gpr41*	*Atgl*	0.863	< 0.001	0.670	0.011	Strong
	Gpr41	*Hsl*	0.853	< 0.001	0.817	< 0.001	Very Strong
	Gpr41	*Cpt1a*	0.734	0.003	0.515	0.06	Moderate
	Gpr41	*Ppargc1a*	0.813	< 0.001	0.275	0.32	Moderate
	Gpr109a	*Atgl*	0.627	0.012	0.676	0.006	Strong
	Gpr109a	*Hsl*	0.597	0.015	0.494	0.052	Moderate
	Gpr109a	*Cpt1a*	0.857	< 0.001	0.787	< 0.001	Very Strong
	Gpr109a	*Ppargc1a*	0.306	0.287	0.398	0.16	Very Weak
